# A Nomogram Based on CT Radiomics and Clinical Risk Factors for Prediction of Prognosis of Hypertensive Intracerebral Hemorrhage

**DOI:** 10.1155/2022/9751988

**Published:** 2022-12-07

**Authors:** Caiyun Fang, Xiao An, Kejian Li, Juntao Zhang, Hui Shang, Tianyu Jiao, Qingshi Zeng

**Affiliations:** ^1^Department of Radiology, The First Affiliated Hospital of Shandong First Medical University, Shandong Provincial Qianfoshan Hospital, Jinan, Shandong 250014, China; ^2^Shandong First Medical University, Shandong Academy of Medical Sciences, Jinan, Shandong 250000, China; ^3^GE Healthcare Precision Health Institution, Shanghai, China

## Abstract

**Purpose:**

To develop and validate a clinical-radiomics nomogram based on clinical risk factors and CT radiomics feature to predict hypertensive intracerebral hemorrhage (HICH) prognosis.

**Methods:**

A total of 195 patients with HICH treated in our hospital from January 2018 to January 2022 were retrospectively enrolled and randomly divided into two cohorts for training (*n* = 138) and validation (*n* = 57) according to the ratio of 7 : 3. All CT radiomics features were extracted from intrahematomal, perihematomal, and combined intra- and perihematomal regions by using free open-source software called 3D slicer. The least absolute shrinkage and selection operator method was used to select the optimal radiomics features, and the radiomics score (Rad-score) was calculated. The relationship between Rad-score, clinical risk factors, and the HICH prognosis was analyzed by univariate and multivariate logistic regression analyses, and the clinical-radiomics nomogram was built. The area under the receiver operating characteristic curve (AUC) and decision curve analysis (DCA) were used to evaluate the performance of the clinical-radiomics nomogram in predicting the prognosis of HICH.

**Results:**

A total of 1702 radiomics features were extracted from the CT images of each patient for analysis. By univariate and stepwise multivariate logistic regression analyses, age, sex, RBC, serum glucose, D-dimer level, hematoma volume, and midline shift were clinical risk factors for the prognosis of HICH. Rad-score and clinical risk factors developed the clinical-radiomics nomogram. The nomogram showed the highest predictive efficiency in the training cohort (AUC = 0.95, 95% confidence interval (CI), 0.92 to 0.98) and the validation cohort (AUC = 0.90, 95% CI, 0.82 to 0.98). The calibration curve indicated that the clinical-radiomics nomogram had good calibration. DCA showed that the nomogram had high applicability in clinical practice.

**Conclusions:**

The clinical-radiomics nomogram incorporated with the radiomics features and clinical risk factors has good potential in predicting the prognosis of HICH.

## 1. Introduction

Hypertensive intracerebral hemorrhage (HICH) is one of the most common types of intracerebral hemorrhage (ICH) [1]. Brain CT scanning is the standard imaging for diagnosing HICH, which is a practical method to determine the location and volume of HICH [2]. On the unenhanced CT image, the hemorrhage mainly showed a high-density mass shadow of hematoma and a rim of hypodensity around the hematoma. The perihematomal hypodensity region is related to various pathophysiological processes, such as cytotoxic edema and neuroinflammation [3]. Previous literature has shown that the perihematomal microenvironment might have crucial biological information and potentially predicting hematoma expansion [4, 5].

Given the high clinical mortality and disability rate of HICH [6], the identification of patients with poor prognoses can help clinicians reach objective opinions on predictable functional outcomes and make better treatment decisions. The so-called CT signs, such as “blend sign,” “island sign,” “black hole sign,” and “swirl sign,” have been proved to help predict hematoma expansion [7–10], whereas these signs have limited in predicting the prognosis of HICH, and these signs are vulnerable to inter- or intraobserver variations [11]. Radiomics is a new research method, which refers to the high-throughput extraction and analysis of a large number of high-dimensional quantitative image features from different modes of medical images [12]. Its advantage is to convert visual image information into deep-seated features for quantitative research [13]. Moreover, radiomics allows multiple imaging features to be studied in parallel, which can provide a combination of features, and the analysis of combined features is more promising than that of single-feature analysis [14]. Although some studies have reported positive results in the analysis of radiomics features [15–17], a new statistical model integrating the combined radiomics features and clinical risk factors has not been developed to predict the prognosis of HICH. Nomogram is a forecasting tool, which can transform the complex regression equation into a visual graph, so as to provide accurate and personalized medical services [18, 19]. In the past, most studies mainly focused on the intrahematomal region, ignoring the perihematomal region [15, 20]. The predictive value of radiomics features in the perihematomal region is not clear.

In the present study, we hypothesized that combining clinical risk factors and CT radiomics features (including radiomics features of intra- and perihematomal regions) could identify HICH patients with poor prognoses. To verify the feasibility of our hypothesis, we extracted the radiomics features based on brain CT and established a clinical-radiomics nomogram integrating radiomics features and clinical risk factors through multivariate logistic regression analysis.

## 2. Materials and Methods

### 2.1. Patients

**Figure 1:**
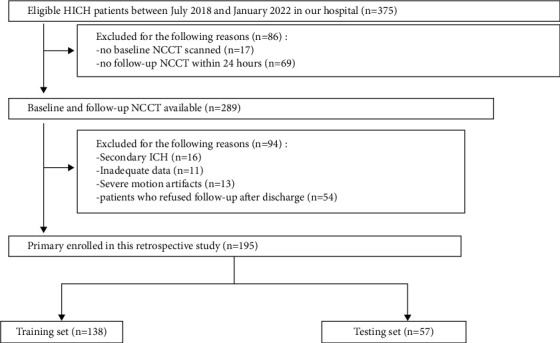
Flow chart for selection of patients with hypertensive intracerebral hemorrhage (HICH).

The local institutional review board approved the retrospective study, and the requirement for informed consent was waived. The CT images and clinical data of HICH patients treated in our hospital from July 2018 to January 2022 were collected. The inclusion criteria were as follows: (1) age >18 years old; (2) a history of hypertension; (3) the first baseline brain CT scanned within 6 hours after the onset of symptoms; (4) brain parenchymal bleeding; (5) complete clinical data. The exclusion criteria were as follows: (1) secondary ICH, such as cerebral aneurysm, trauma, arteriovenous malformation, tumor, or hemorrhagic infarction; (2) patients with anticoagulant-associated ICH; (3) motion artifacts on CT images; (4) patients who refused follow-up after discharge. The flow chart is shown in Figure 1.

In addition, the following relevant clinical information was obtained through the patient's inpatient medical record system and the emergency medical record system: (1) clinical data included age, sex, systolic blood pressure, diastolic blood pressure, smoking history, drinking history, and diabetes history; (2) admission laboratory parameters included platelet count, white blood cell count, red blood cell (RBC) count, lymphocyte count, monocyte count, hemoglobin, serum glucose, and D-dimer level.

### 2.2. Functional Outcome Assessment

90 days after discharge, the patients were followed up by standardized telephone interviews, and the clinical functional outcome of HICH patients was assessed by Glasgow Outcome Scale (GOS). Referring to a previous study [21], we classified the prognosis of patients into two categories: unfavorable outcome (GOS 1, death; GOS 2, persistent vegetative state; GOS 3, severe disability) and favorable outcome (GOS 4, moderate disability; GOS 5, return to normal life).

### 2.3. CT Image Acquisition and Data Collection

All patients underwent brain CT with GE Optima CT660 64 row spiral scanner. Patients were placed in the supine position. The head was placed in the head frame of the examination table, and the two external ear holes were equidistant from the table. At 120 kV tube voltage, 300 mA tube current, 512 × 512 matrix, and 5 mm slicer thickness, scanning was performed in a continuous cross section from the skull base to the skull top after taking a positioning image.

According to the CT images, the CT plain scan signs, such as hematoma volume, hematoma location, the degree of midline shift, whether it broke into the cerebral ventricle, herniation, and ventricular entrapment were obtained. The imaging data were independently evaluated by a senior radiologist who was blinded to the clinical information. The hematoma volume was calculated by using formula *A* × *B* × *C*/2 [22]. *A* is the longest diameter on the largest hematoma slice, *B* is the most significant diameter perpendicular to *A*, and *C* is the number of bleeding layers in CT multiplied by the slice thickness. The degree of midline shift was determined according to previous literature [23].

### 2.4. Image Segmentation and Feature Extraction

**Figure 2:**
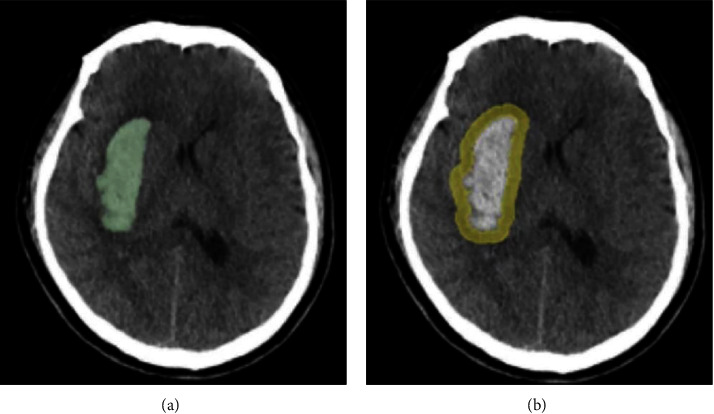
An example of segmentation of the region of interest (ROI) in hypertensive intracerebral hemorrhage. (a) The intrahematomal ROI (intra-ROI) was manually segmented based on the contour of hematoma; (b) the perihematomal ROI (peri-ROI) was automatically reconstructed based on the contour of the intra-ROI using the “dilation” algorithm.

The brain CT images of 30 patients were randomly selected to evaluate the interobserver agreement of feature extraction. Two experienced radiologists (readers 1 and 2) independently and manually completed the hematoma contour blinded to clinical data. Reproducibility of interobserver for drawing region of interest (ROI) was assessed by intraclass correlation coefficient (ICC). ICC value above 0.75 was considered to have good consistency, and all the remaining images were completed by Reader 1. In addition, we also captured the information around the hematoma from the surrounding area 6 mm away from the hematoma surface. According to the contour of the intrahematomal ROI (intra-ROI), we used the the “dilation” algorithm to automatically reconstruct the perihematomal ROI (peri-ROI) and obtained a ring of brain parenchyma around the hematoma.  Figure 2 shows an example of drawing intra-ROI and peri-ROI.

All radiomics features were extracted by using free open-source software called 3D slicer (version 4.13, https://www.slicer.org). A total of 851 features were extracted from each ROI, which can be summarized into the following four groups: 14 volume and shape features (2D and 3D), 18 first-order features, 75 texture features, and 744 wavelet transform features. Three groups of features were obtained from the intra-ROI, peri-ROI, and their combined ROI (intra-ROI + peri-ROI).

### 2.5. Radiomics Feature Screening and Rad-Score Building

The minimum-redundancy maximum-relevance (mRMR) and the least absolute shrinkage and selection operator (LASSO) method were used for feature selection. Initially, mRMR was applied to eliminate redundant and irrelevant features. Then, the LASSO algorithm was conducted to select the optimized feature subset using ten-fold cross-validation to build the final model. The radiomics score (Rad-score) was calculated for each patient by a linear combination of selected features weighted by their respective coefficients. Based on the selected features of intra-ROI, peri-ROI, and their combined ROI, three radiomics models, which were intrahematomal-based model (intra-model), perihematomal ring-based model (peri-model), and combined model, were then established. The workflow of radiomics analysis of hematoma is shown in Supplementary Figure 1.

### 2.6. Development of Clinical and Clinical-Radiomics Model

Univariate and multivariate logistic regression analyses were used to analyze the relationship between the prognosis and clinical characteristics of HICH and screen out the clinical risk factors in developing a clinical model for the prognosis. Moreover, clinical risk factors and radiomics features (including radiomics features of intra- and perihematomal areas) were merged by a multivariate logistic regression to develop the clinical-radiomics model. For the visualization and clinical application of the model, the model was displayed by nomogram.

### 2.7. Statistical Analysis

Statistical analysis and data processing were performed using the R programming language (version 4.1.0, https://www.programmingr.com). Comparisons between sets were performed by the independent sample *t*-test or Mann–Whitney *U* test for continuous variables and the Chi-square test or Fisher's exact test for categorical variables. The accuracy of each model in predicting the prognosis of HICH was evaluated by the area under the receiver operating characteristic (ROC) curve (AUC), and Delong test was utilized to compare the AUC difference between nomogram and clinical model. The application value of the nomogram in the training cohort and validation cohort was assessed by decision curve analysis (DCA). A two-tailed *P*-value <0.05 represented a statistical significance.

## 3. Results

### 3.1. Clinical Characteristics

A total of 195 HICH patients were enrolled consecutively, including 126 males and 69 females, with an average age of 59.32 ± 13.11 years (range 19–90 years). Patients were divided into training cohort (*n* = 138) and validation cohort (*n* = 57) according to the ratio of 7 : 3. During the 90-day follow-up using the GOS score, 69 patients (35.4%) had a good prognosis, and 126 patients (64.6%) had a poor prognosis.  Table 1 summarizes the clinical characteristics of patients in the training and validation cohorts.

### 3.2. Radiomics Feature Selection and Rad-Score Construction

**Figure 3:**
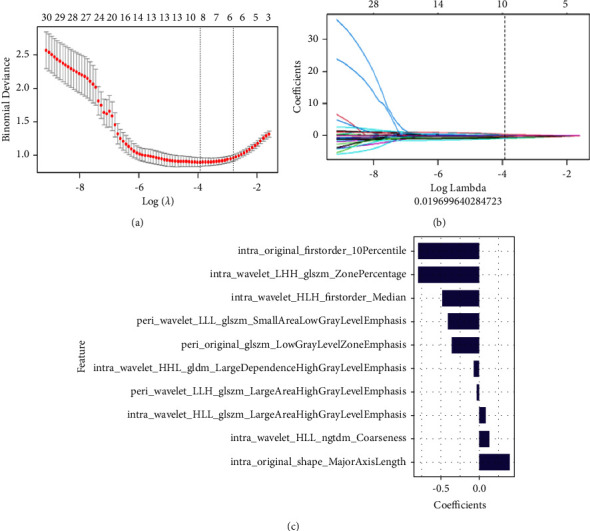
Texture feature selection using the least absolute shrinkage and selection operator (LASSO) method. (a) The optimal tuning parameter (*λ*) was selected using 10-fold cross-validation in the LASSO regression model; (b) LASSO regression coefficient distribution; (c) optimal radiomics feature combination and its correlation coefficient.

First, 1702 radiomics features were extracted from the depicted ROI, including manually segmented intra-ROI and automatically segmented peri-ROI. The interobserver ICC ranged from 0.751 to 0.997, so these features had good repeatability. Next, 11 optimal radiomics features were selected from intra-ROI and peri-ROI by mRMR and LASSO, respectively, and then, incorporating intra- and peri-ROI features, ten radiomics features (seven from intra-ROI and three from peri-ROI) were selected (Figure 3). Finally, the Rad-score was calculated for each patient using the formula provided in Supplementary Table 1 from the selected features.

### 3.3. Establishment of Radiomics, Clinical, and Clinical-Radiomics Model

Three radiomics models for predicting the prognosis of HICH were built based on radiomics features of intra-ROI, peri-ROI, and their combined ROI, respectively. We drew the ROC curves to compare the predictive accuracy of three radiomics models. The specific results are shown in Supplementary Figure 2. In the validation cohort, the AUC of the combined model was 0.90 (95% confidence interval (CI), 0.82 to 0.98), which was superior to the intra-model (AUC = 0.88, 95% CI, 0.79 to 0.97) and the peri-model (AUC = 0.82, 95% CI, 0.71 to 0.93).

By univariate and stepwise multivariate logistic regression analyses, age, sex, RBC, serum glucose, D-dimer level, hematoma volume, and midline shift were independent predictors for the prognosis of HICH (Table 2). Then, seven clinical risk factors were used to establish the clinical model. In addition, because the combined radiomics model has the best performance, the clinical-radiomics model was further established by integrating the combined Rad-score and clinical risk factors.

### 3.4. Performance Evaluation of Predictive Model and Development of Nomogram

**Figure 4:**
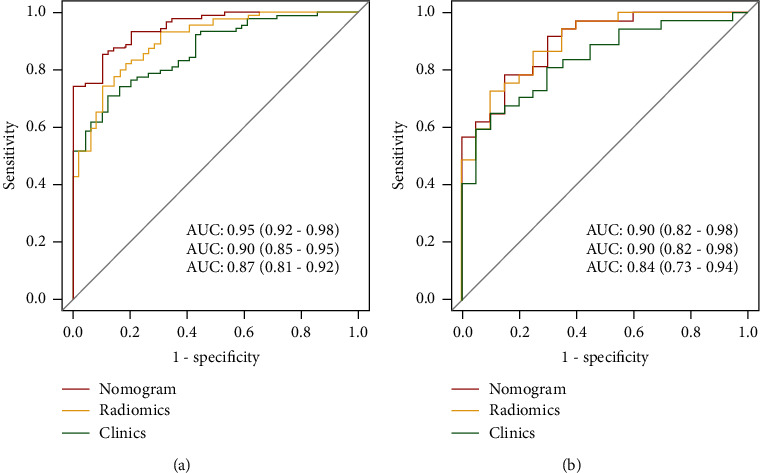
Receiver operating characteristic curves of the nomogram, radiomics model, and clinical model to predict the prognosis of HICH ((a) is the training queue and (b) is the validation queue).

**Figure 5:**
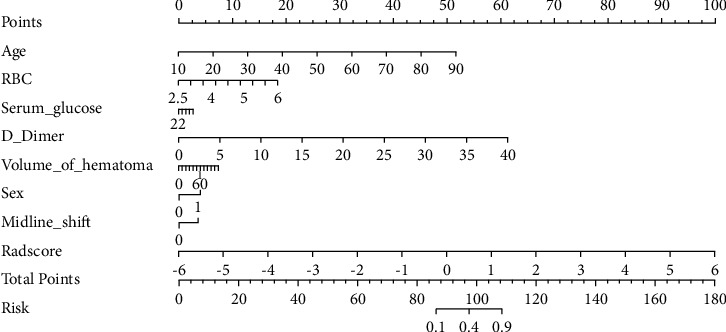
Clinical-radiomics nomogram. The nomogram was composed of Rad-score, age, sex, RBC, serum glucose, D-dimer, volume of hematoma, and midline shift. In sex, 0 indicated male and 1 indicated female; in midline shift, 0 showed no and 1 showed yes.

Figure 4 shows the performance of the clinical model, combined radiomics model, and clinical-radiomics model in predicting the prognosis of HICH in the training and validation queues. The clinical-radiomics model showed the highest discrimination in the training cohort in identifying patients with excellent and poor prognoses, with an AUC of 0.95 (95% CI, 0.92 to 0.98). The AUC of the clinical-radiomics model was significantly higher than that of the clinical model (AUC = 0.87, 95% CI, 0.81 to 0.92) and the combined radiomics model (AUC = 0.90, 95% CI, 0.85 to 0.95). In the validation cohort, the AUC of the clinical-radiomics model of 0.90 (95% CI, 0.82 to 0.98) was superior to that of the clinical model (AUC = 0.84, 95% CI, 0.73 to 0.94). The clinical-radiomics model showed the best performance in predicting the prognosis of HICH, surpassing all the other models, and had the highest prediction accuracy. Moreover, the ROC comparison verified by Delong test showed statistical significance between nomogram model and clinical model (*Z* = 3.56, *P* < 0.01), suggesting that the clinical predicted net return of nomogram was higher than that of clinical model. Based on this best model, we generated a visualized clinical-radiomics nomogram (Figure 5).

### 3.5. Clinical Application

**Figure 6:**
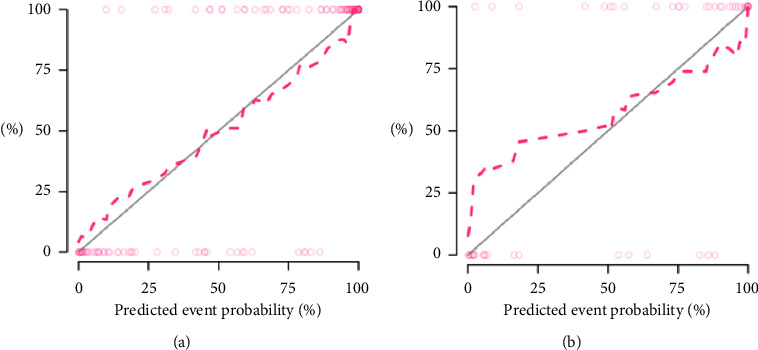
Calibration curves for the clinical-radiomics nomogram in training cohort (a) and validation cohort (b).

**Figure 7:**
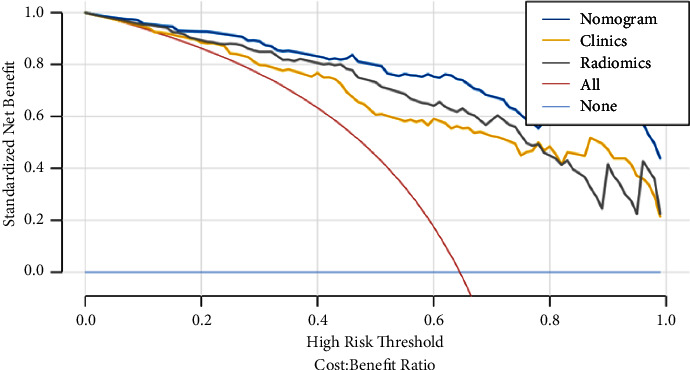
Clinical decision curve of the three models. The vertical axis is the net benefit rate, and the horizontal axis is the probability threshold. DCA shows that the nomogram (blue line) adds more benefit than either all treatment schemes (red line) or no treatment schemes (light blue line) in predicting the prognosis of HICH when the threshold probability ranges from 0.1 to 1.0. Moreover, compared with the radiomics model (grey line) and clinical model (orange line), the nomogram model (blue line) achieved the highest net benefit.

The calibration curve showed that the clinical-radiomics nomogram had a good consistency and high calibration degree in predicting the prognosis of HICH and the actual results (Figure 6). Moreover, the DCA curves of clinical-radiomics nomogram, clinical model, and combined radiomics model showed that clinical-radiomics nomogram had more excellent clinical utility, which indicated that the nomogram was a reliable clinical tool. In addition, DCA showed that these models were better than the “all treatment” and “no treatment” indexes in the training cohort in predicting the prognosis of HICH (Figure 7).

## 4. Discussion

HICH is a common disease in neurosurgery, accounting for 70%–80% of all cases of ICH. The prognosis is poor, which will endanger the lives of patients and seriously affect people's health and quality of life [24]. Early and accurate prediction of the prognosis of HICH patients is the key to personalized treatment of HICH. In this study, to identify patients with poor prognoses, we developed and validated the clinical-radiomics nomogram based on the radiomics features and clinical risk factors. The nomogram showed good performance in training and validation cohorts and was an easy-to-use personalized decision-making tool.

In terms of clinical characteristics, through multivariate logistic regression analysis, our study found that age, sex, RBC, serum glucose, D-dimer level, hematoma volume, and midline shift were the clinical risk factors for predicting the prognosis of HICH. Previous studies have shown that age, sex, hematoma volume, and midline shift can be used to predict functional outcomes in ICH patients [23, 25, 26], which is consistent with our results. In addition, we also found that the risk of poor prognosis at 90 days was related to RBC, serum glucose, and D-dimer level. Low RBC levels are associated with poor ICH prognoses, which may be partly due to impaired cerebral oxygenation [27]. Béjot et al. [28] found that admission hyperglycemia was associated with 1-month mortality and poor functional recovery at discharge. Moreover, basic studies have also confirmed the effect of hyperglycemia on early hematoma expansion, mainly manifested in neuron death, angiogenic brain edema, and aggravation of blood-brain barrier damage [29, 30]. Zhou et al. [31] indicated that elevated plasma D-dimer levels after ICH were associated with mortality and poor functional outcomes. The increase in D-dimer level is related to progressive bleeding injury, which may reflect the disturbance of cerebral microcirculation and systemic hypercoagulability [32]. Based on these clinical risk factors, we developed a clinical model to predict the prognosis of HICH patients. The diagnostic effect of this model was good, with AUCs of 0.87 and 0.84 in the training and validation cohorts, respectively.

Radiomics methods have great potential in promoting clinical decision-making by improving the accuracy of clinical diagnosis, prognosis prediction, and treatment response [33]. In the present study, we attempted to apply a novel combined intra- and perihematomal radiomics method to predict the prognosis of HICH. Compared with the research of Xu et al. [15], we not only analyzed the radiomics features of the intra-ROI but also explored the radiomics features of the peri-ROI. Previous study has shown that the perihematomal microenvironment is related to the pathophysiological process of hematoma expansion and may provide some potential predictive information [34]. In our study, three of the ten best features in the combined radiomics model were from peri-ROI, which indicated that the perihematomal region might provide incremental information. In addition, our findings showed that in the validation cohort, the AUC of the combined radiomics model, incorporating intra- and peri-ROI features, was 0.90 (95% CI, 0.82–0.98), which was better than the single intra- and perimodel, and yielded the overall best prediction performance. This finding indicated that the radiomics features of the peri-ROI might have potential value and deserved further exploration.

We used machine learning methods (radiomics features) to evaluate the characteristics of the hematoma itself and around the hematoma in order to better assess the heterogeneity of the hematoma. Rad-score can be used to quantitatively reflect the characteristics of the hematoma itself after radiomics analysis, and it can be concluded through the logistic regression analysis that Rad-score is an independent variable of HICH prognosis. By adding Rad-score to the clinical model, a clinical-radiomics nomogram was developed to promote further clinical application and accurately predict the prognosis of HICH. Compared with other models, this nomogram has further improved the performance of predicting the prognosis of HICH and achieved higher accuracy. The AUCs of the training and validation cohorts were 0.95 and 0.90, respectively, which outperformed the single clinical characteristics and the radiomics features. The doctor can add the scores of each prediction variable and get the total score according to the individual differences of patients, so as to better help clinical decision-making and enable clinicians to develop personalized treatment plans for HICH patients. In addition, the calibration curve and DCA curve showed that the nomogram had good consistency and potential clinical applicability, and the maximum benefit was obtained under all thresholds.

However, the present research still has some limitations. Firstly, only the radiomics of the 6 mm perihematomal area was analyzed. The predictive ability of other perihematomal region radiomics models with different distances to the prognosis of HICH patient needs to be further analyzed and studied. Secondly, this study is a single-center retrospective study, and the sample size is relatively small, which inevitably has some deviations; hence, large sample prospective and external validation studies are required.

## 5. Conclusions

In conclusion, this study established a clinical-radiomics nomogram, composed of radiomics features (including radiomics features of intra-ROI and peri-ROI) and clinical risk factors to identify HICH patients with poor prognoses. It can assist doctors in making clinical treatment decisions for patients with poor prognoses. Moreover, the clinical-radiomics nomogram shows potential value in precision medicine and designs personalized treatment strategies to better achieve personalized precision treatment.

## Figures and Tables

**Table 1:** The clinical characteristics of the training cohort and the validation cohort.

Characteristics	Training cohort (*n* = 138)		*P*	Validation cohort (*n* = 57)		*P*
	Favorable outcome (*n* = 49)	Unfavorable outcome (*n* = 89)		Favorable outcome (*n* = 20)	Unfavorable outcome (*n* = 37)	
Sex (*n*, %)			0.037^*∗*^			0.604
Female	10 (20.4)	35 (39.3)		13 (65.0)	20 (54.1)	
Male	39 (79.6)	54 (60.7)		7 (35.0)	17 (45.9)	
Age (year)	51.6 ± 11.6	63.7 ± 12.0	<0.001^*∗*^	53.9 ± 11.0	62 ± 13.3	0.020^*∗*^
SBP (mmHg)	165.8 ± 27.3	163.6 ± 25.1	0.637	158.7 ± 25.0	167.6 ± 33.2	0.294
DBP (mmHg)	98.1 ± 19.2	96.0 ± 16.5	0.493	93.5 ± 13.4	93.4 ± 16.5	0.998
Smoking (*n*, %)			1.000			0.623
No	31 (63.3)	56 (62.9)		12 (60.0)	26 (70.3)	
Yes	18 (36.7)	33 (37.1)		8 (40.0)	11 (29.7)	
Drinking (*n*, %)			0.223			0.544
No	22 (44.9)	51 (57.3)		10 (50.0)	23 (62.2)	
Yes	27 (55.1)	38 (42.7)		10 (50.0)	14 (37.8)	
Diabetes (*n*, %)			0.050^*∗*^			0.482
No	46 (93.9)	71 (79.8)		17 (85.0)	27 (73.0)	
Yes	3 (6.1)	18 (20.2)		5 (15.0)	10 (27.0)	
Monocyte count (10^9^/L)	0.4 ± 0.2	0.5 ± 0.3	0.084	0.6 ± 0.2	0.5 ± 0.3	0.446
WBC (10^9^/L)	10.3 ± 4.0	11.9 ± 4.9	0.048^*∗*^	11.3 ± 3.9	12.0 ± 4.9	0.571
Lymphocyte count (10^9^/L)	1.3 ± 0.9	1.3 ± 0.9	0.760	1.3 ± 0.5	1.2 ± 0.8	0.493
RBC (10^12^/L)	4.8 ± 0.6	4.5 ± 0.6	0.037^*∗*^	4.6 ± 0.6	4.7 ± 0.7	0.648
Platelet count (10^9^/L)	224.6 ± 60.6	229.0 ± 78.8	0.732	230.5 ± 49.8	227.4 ± 74.6	0.866
Hemoglobin (g/L)	143.1 ± 22.2	137.9 ± 18.8	0.150	142.5 ± 17.0	142.4 ± 17.9	0.986
Serum glucose (mmol/L)	7.5 ± 2.9	9.1 ± 3.6	0.007^*∗*^	8.3 ± 4.0	10.5 ± 3.7	0.031^*∗*^
D-dimer (mg/L)	0.5 ± 0.5	1.9 ± 4.3	0.022^*∗*^	0.6 ± 0.4	1.8 ± 2.0	0.006^*∗*^
Volume of hematoma (mL)	15.2 ± 13.2	30.8 ± 23.1	<0.001^*∗*^	15.1 ± 8.8	30.5 ± 22.7	0.003^*∗*^
Location (*n*, %)			0.698			0.197
Basal ganglia	41 (83.7)	69 (77.5)		17 (85.0)	25 (67.6)	
Lobe	6 (12.2)	12 (13.5)		2 (10.0)	2 (5.4)	
Brainstem	0 (0.0)	1 (1.1)		0 (0.0)	6 (16.2)	
Cerebellum	2 (4.1)	7 (7.9)		1 (5.0)	4 (10.8)	
Intraventricular hemorrhage (*n*, %)			0.059			0.064
No	31 (63.3)	40 (44.9)		14 (70.0)	15 (40.5)	
Yes	18 (36.7)	49 (55.1)		6 (30.0)	22 (59.5)	
Midline shift (*n*, %)			0.006^*∗*^			0.833
No	33 (67.3)	37 (41.6)		10 (50.0)	16 (43.2)	
Yes	16 (32.7)	52 (58.4)		10 (50.0)	21 (56.8)	
Herniation			0.552			0.191
No	46 (93.8)	81 (91.0)		20 (100.0)	34 (91.9)	
Yes	3 (6.2)	8 (9.0)		0 (0.0)	3 (8.1)	
Ventricular entrapment			0.061			0.105
No	43 (87.8)	66 (72.2)		19 (95.0)	31 (83.8)	
Yes	6 (12.2)	23 (25.8)		1 (5.0)	6 (16.2)	
Rad-score	−0.6 (−1.3, 0.3)	1.6 (0.6, 3.1)	<0.001^*∗*^	−1.3 (−1.7,−0.2)	1.3 [0.2, 2.4]	<0.001^*∗*^

*Notes*. ^*∗*^*P* < 0.05, indicating that the difference was statistically significant. SBP, systolic blood pressure; DBP, diastolic blood pressure; WBC, white blood cell; RBC, red blood cell; Rad-score, radiomics score.

**Table 2:** Univariate and multivariate logistic analyses of prognostic risk factors for HICH.

Variable	Univariate logistic analysis		Multivariate logistic analysis	
	OR (95% CI)	*P*	OR (95% CI)	*P*
Age	1.09 (1.05, 1.14)	<0.001	1.14 (1.06, 1.22)	<0.001
WBC	1.08 (0.99, 1.18)	0.054	NA	NA
RBC	0.53 (0.29, 0.98)	0.042	2.50 (0.87, 7.14)	0.087
Serum glucose	1.19 (1.03, 1.38)	0.012	0.98 (0.83, 1.17)	0.832
D-dimer	2.41 (1.31, 4.45)	0.004	1.36 (0.80, 2.32)	0.258
Volume of hematoma	1.05 (1.02, 1.08)	<0.001	1.01 (0.97, 1.06)	0.570
Sex	2.52 (1.11, 5.70)	0.025	2.25 (0.57, 8.92)	0.248
Midline shift	2.89 (1.39, 6.02)	0.004	2.05 (0.58, 7.25)	0.263
Rad-score	4.40 (2.59, 7.47)	<0.001	5.33 (2.88, 11.93)	<0.001

*Notes*. WBC, white blood cell; RBC, red blood cell; Rad-score, radiomics score; OR, odds ratio; CI, confidence interval; NA, not available.

## Data Availability

The data used to support the findings of this study are available from the corresponding author upon request.

## Supplementary Materials

Supplementary Materials Supplementary Figure 1: the workflow of the radiomics analysis of hematoma. Supplementary Table 1: radiomics features selected by mRMR and LASSO methods for establishing radiomics signatures. Supplementary Figure 2: ROC curves of the radiomics features of the intrahematomal area (A and B), perihematomal area (C and D), and intrahematomal + perihematomal area (E and F) in the training cohort and the validation cohort.
