# Supplementary material for: A Nomogram Based on CT Radiomics and Clinical Risk Factors for Prediction of Prognosis of Hypertensive Intracerebral Hemorrhage
Source: Comput Intell Neurosci. 2022 Dec 7;2022:9751988. doi: 10.1155/2022/9751988 (PMC9750770; doi:10.1155/2022/9751988)
Supplement: Supplementary Materials — Supplementary Figure 1: the workflow of the radiomics analysis of hematoma. Supplementary Table 1: radiomics features selected by mRMR and LASSO methods for establishing radiomics signatures. Supplementary Figure 2: ROC curves of the radiomics features of the intrahematomal area (A and B), perihematomal area (C and D), and intrahematomal + perihematomal area (E and F) in the training cohort and the validation cohort. [file 9751988.f1.docx]

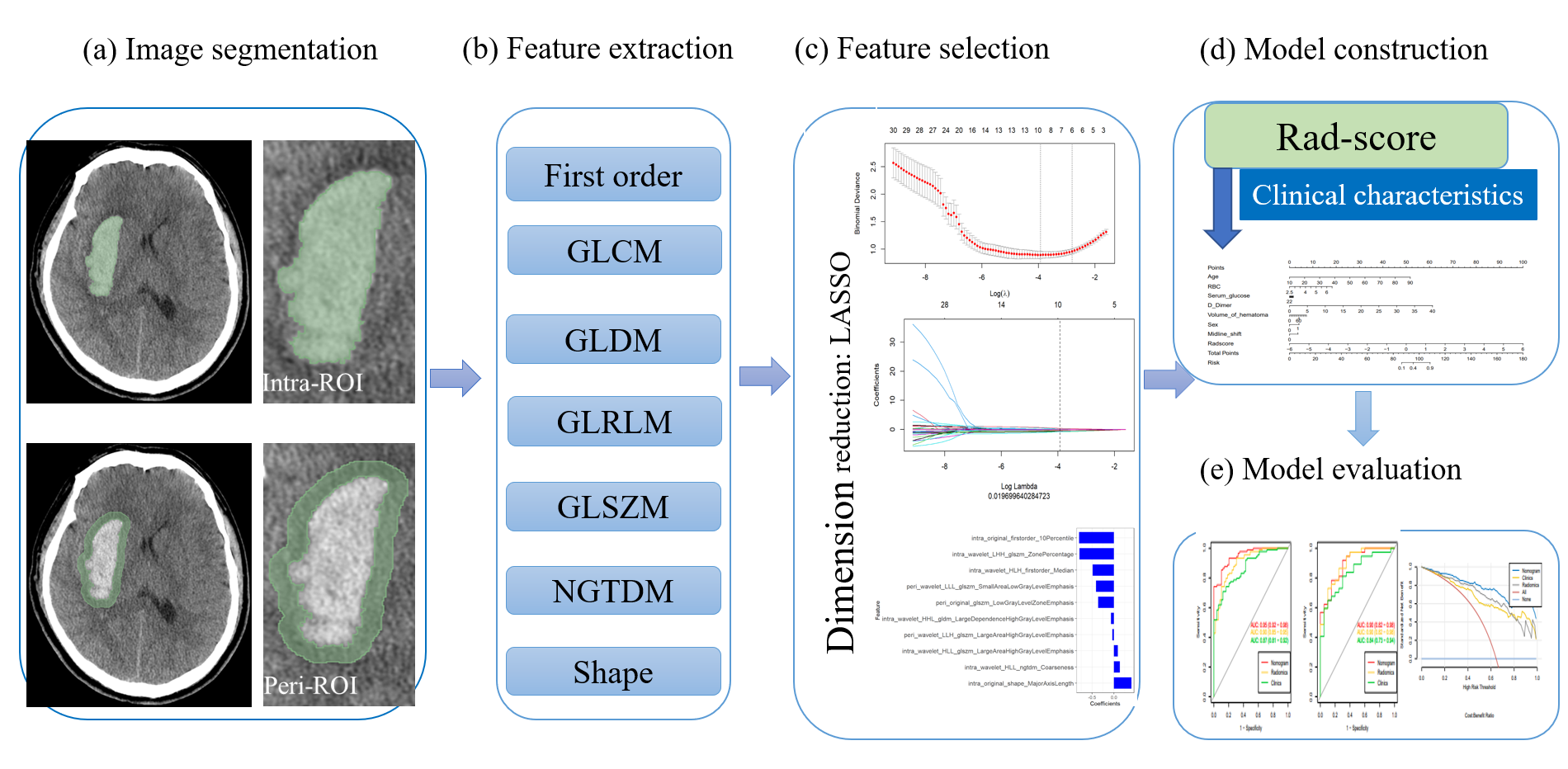


Supplementary Figure 1: The workflow of the radiomics analysis of hematoma. Notes: intra-ROI= the intrahematomal region of interest (ROI); peri-ROI: the perihematomal ROI; GLCM = the gray-level cooccurrence matrix; GLDM= the gray level dependence matrix; GLRLM=the gray level run length matrix; GLSZM = the gray-level size zone matrix; NGTDM= the neighborhood gray tone difference matrix; LASSO = least absolute shrinkage and selection operator; Rad-score= the radiomics score.

| Supplementary Table 1: Radiomics features selected by mRMR and LASSO method for establishing radiomics signatures. | | |
| --- | --- | --- |
| Radiomics signature (Number of features) | Features | Coefficient |
| Intrahematomal regions (11) | wavelet_LLL_glszm_ZoneEntropy | 0.290 |
|  | wavelet_HHH_ngtdm_Strength | -0.255 |
|  | original_firstorder_10Percentile | -0.836 |
|  | wavelet_LHL_gldm_LargeDependenceHighGrayLevelEmphasis | 0.122 |
|  | wavelet_LLL_glszm_SmallAreaLowGrayLevelEmphasis | -0.099 |
|  | wavelet_HLH_firstorder_Median | -0.344 |
|  | wavelet_HLL_glszm_LargeAreaHighGrayLevelEmphasis | 0.133 |
|  | original_glcm_Correlation | 0.062 |
|  | wavelet_LHL_glrlm_RunPercentage | -0.103 |
|  | original_glszm_GrayLevelNonUniformity | 0.490 |
|  | wavelet_LLH_gldm_DependenceVariance | 0.391 |
| Perihematomal regions (11) | wavelet_LLL_glrlm_RunLengthNonUniformity | 0.341 |
|  | wavelet_HHH_ngtdm_Strength | -0.089 |
|  | wavelet_LLL_glszm_SmallAreaLowGrayLevelEmphasis | -0.129 |
|  | original_firstorder_10Percentile | -0.121 |
|  | original_shape_MajorAxisLength | 0.289 |
|  | wavelet_LHL_glrlm_ShortRunEmphasis | -0.020 |
|  | wavelet_LHH_firstorder_Skewness | 0.151 |
|  | wavelet_LHL_glcm_InverseVariance | -0.355 |
|  | wavelet_LHL_glcm_ClusterTendency | -0.090 |
|  | wavelet_LLL_glrlm_LongRunLowGrayLevelEmphasis | -0.148 |
|  | wavelet_HLL_firstorder_10Percentile | -0.176 |
| Intrahematomal regions + perihematomal regions (10) | intra_original_firstorder_10Percentile | -0.796 |
|  | intra_wavelet_HLL_ngtdm_Coarseness | 0.135 |
|  | peri_wavelet_LLH_glszm_LargeAreaHighGrayLevelEmphasis | -0.036 |
|  | peri_wavelet_LLL_glszm_SmallAreaLowGrayLevelEmphasis | -0.408 |
|  | intra_wavelet_LHH_glszm_ZonePercentage | -0.784 |
|  | intra_wavelet_HLH_firstorder_Median | -0.484 |
|  | intra_original_shape_MajorAxisLength | 0.400 |
|  | intra_wavelet_HHL_gldm_LargeDependenceHighGrayLevelEmphasis | -0.067 |
|  | intra_wavelet_HLL_glszm_LargeAreaHighGrayLevelEmphasis | 0.091 |
|  | peri_original_glszm_LowGrayLevelZoneEmphasis | -0.357 |

Ten radiomics features (seven from intra-ROI and three from peri-ROI) were selected from 1702 (851×2）texture features by using the LASSO regression model.

These features were included in the Rad-score, which is calculated by using the following formula:

"Radscore=-0.796*intra_original_firstorder_10Percentile+0.135*intra_wavelet_HLL_ngtdm_Coarseness+-0.036*peri_wavelet_LLH_glszm_LargeAreaHighGrayLevelEmphasis+-0.408*peri_wavelet_LLL_glszm_SmallAreaLowGrayLevelEmphasis+-0.784*intra_wavelet_LHH_glszm_ZonePercentage+-0.484*intra_wavelet_HLH_firstorder_Median+0.4*intra_original_shape_MajorAxisLength+-0.067*intra_wavelet_HHL_gldm_LargeDependenceHighGrayLevelEmphasis+0.091*intra_wavelet_HLL_glszm_LargeAreaHighGrayLevelEmphasis+-0.357*peri_original_glszm_LowGrayLevelZoneEmphasis + 0.958"

(A)
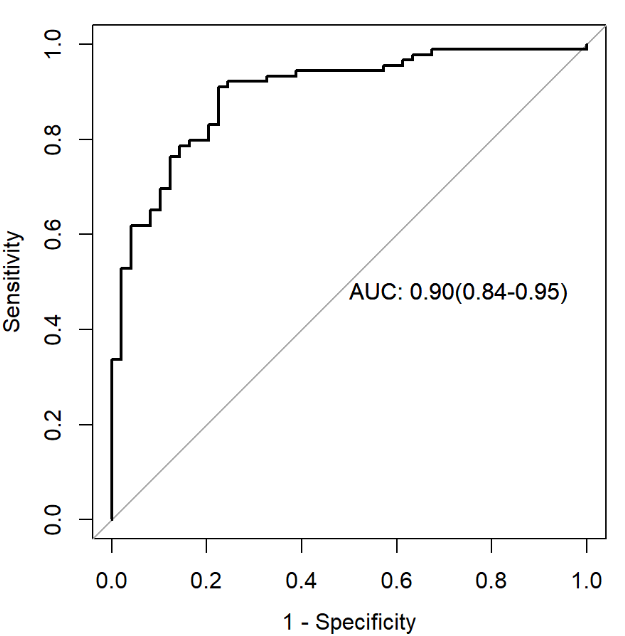
(B)
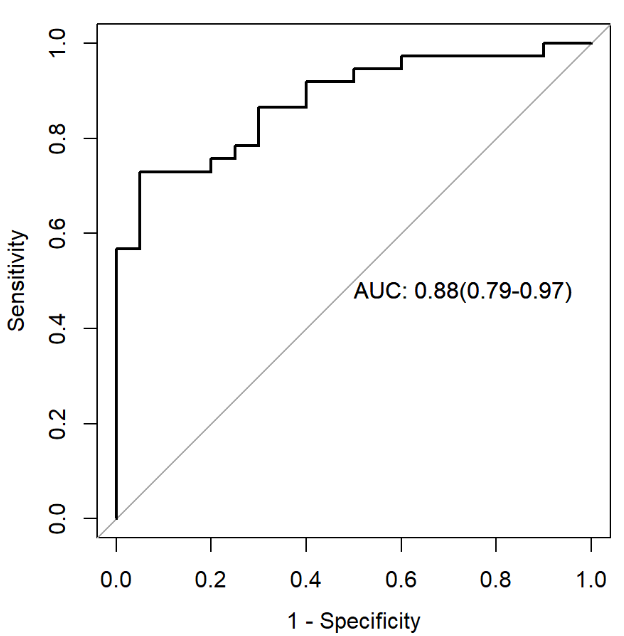
(C)
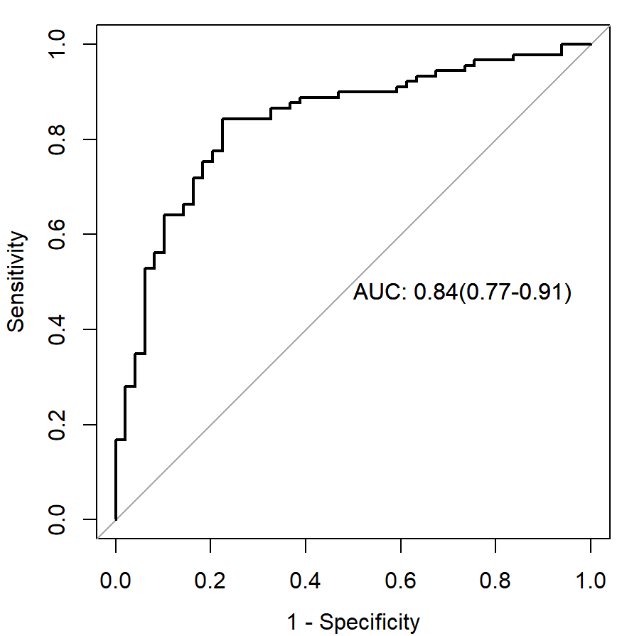


(D)
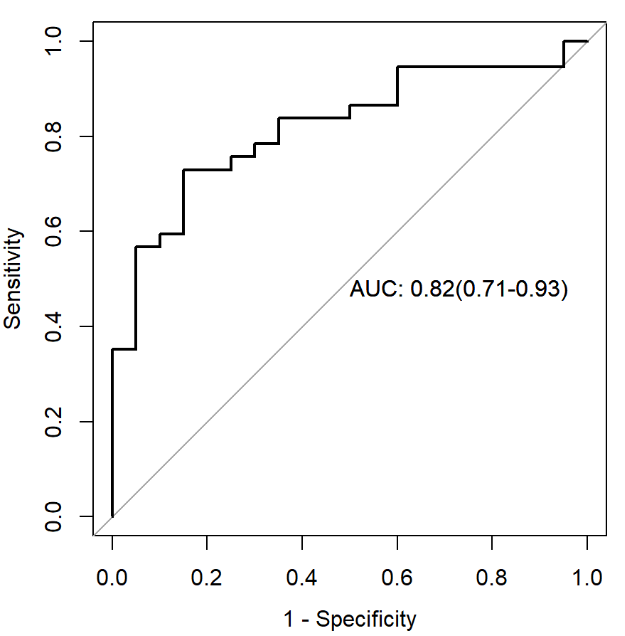
(E)
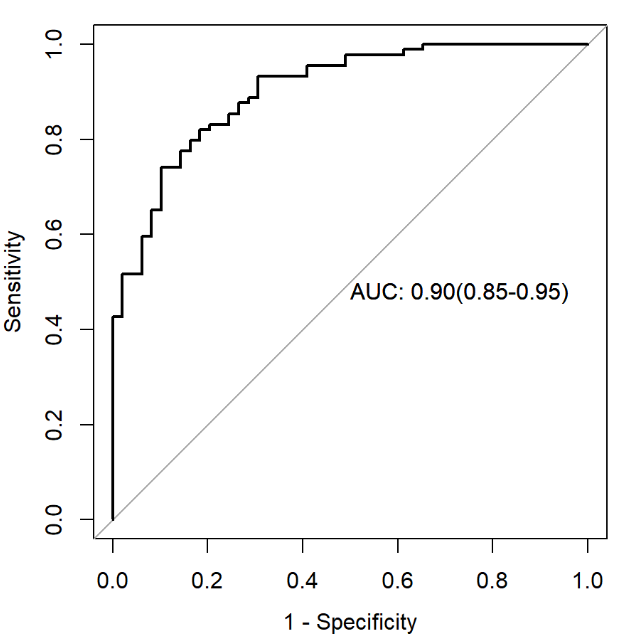
(F)
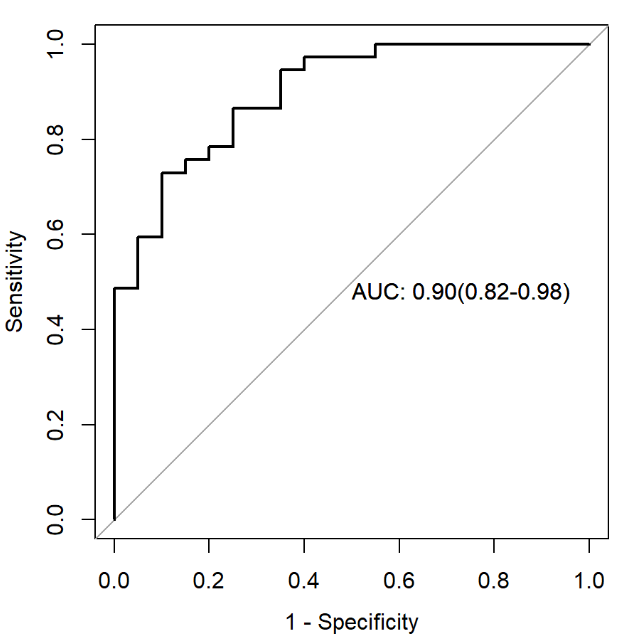


Supplementary Figure 2: ROC curves of the radiomics features of the intrahematomal area (A and B), perihematomal area (C and D) and intrahematomal + perihematomal area (E and F) in the training cohort and the validation cohort.
